# Effects of oral Ketone Ester on brain metabolism and cognition: a randomized controlled trial

**DOI:** 10.1002/alz70859_097825

**Published:** 2025-12-25

**Authors:** Apostolos Manolopoulos, Konstantinos Avgerinos, Qinghua Chen, Josephine M. Egan, Dimitrios Kapogiannis

**Affiliations:** ^1^ National Institute on Aging, Baltimore, MD USA; ^2^ Wayne State University, Detroit, MI USA

## Abstract

**Background:**

Ketone bodies serve as an alternative energy source and signaling molecules that may improve neuronal cellular processes, reduce excitotoxicity, and compensate for glucose hypometabolism in Alzheimer’s disease (AD). Animal studies have demonstrated that exogenous Ketone Ester (KE) administration enhances cognition and reduces brain amyloid and phosphorylated tau. We conducted a double‐blind randomized controlled trial to provide proof‐of‐concept for KE’s target engagement and pro‐cognitive effects in individuals with metabolic syndrome, a population at higher risk for AD.

**Method:**

Fifty cognitively intact participants over 55 years old with metabolic syndrome were randomized (1:1) to receive either 25 g of oral KE or an isocaloric placebo (dextrose) thrice daily for four weeks. The primary outcome was the brain concentration of β‐hydroxybutyrate (βHB) by Magnetic Resonance Spectroscopy (MRS). Secondary outcomes included other brain metabolite concentrations and cognitive performance assessed by the Preclinical Alzheimer Cognitive Composite (PACC) and the NIH Toolbox. Linear mixed models were fitted with Group (KE/Placebo), Week (0/4), Drink (Before/After), and their interactions as fixed effects, subjects as random effects, and covariates for age and education.

**Result:**

KE increased βHB concentration in the brain (p < 0.001), confirming brain penetrance and target engagement. Serum and urine βHB levels also increased following KE administration. Additionally, KE reduced brain glutamate levels (p < 0.001), suggesting decreased glutamatergic excitatory activity, while aspartate levels decreased with both KE and placebo (both p = 0.01). Logical memory improved for both immediate verbatim and gist recall in both groups (indicating practice effects), with trends favoring KE (Chisq_Group*Week_ = 3.77, p = 0.052 and Chisq_Group*Week_ = 3.18, p = 0.07, respectively). Within‐group analyses showed improvements in delayed verbatim recall and digit symbol substitution only with KE. Only mild gastrointestinal adverse events were reported with KE.

**Conclusion:**

Oral KE supplementation successfully induced brain ketosis, confirming target engagement, and reduced brain glutamate levels, suggesting modulation of hyperexcitability and potential neuroprotection. These metabolic changes were accompanied by improvements in memory and executive function, highlighting KE’s potential for AD prevention.

